# Self-Harming and Sense of Agency in Patients With Borderline Personality Disorder

**DOI:** 10.3389/fpsyt.2020.00449

**Published:** 2020-05-29

**Authors:** Livia Colle, Dize Hilviu, Roberta Rossi, Francesca Garbarini, Carlotta Fossataro

**Affiliations:** ^1^Psychology Department, University of Turin, Turin, Italy; ^2^Unit of Psychiatry, IRCCS Istituto Centro San Giovanni di Dio Fatebenefratelli, Brescia, Italy; ^3^MANIBUS Lab, Psychology Department, University of Turin, Turin, Italy

**Keywords:** borderline personality disorder, self-harming behaviors, sensory attenuation, sense of agency, dissociation

## Abstract

Self-harm is considered a pervasive problem in several psychopathologies, and especially in Borderline Personality Disorder (BPD). Self-harming behaviors may be enacted for many purposes for example to regulate emotions and to reduce dissociation. BPD patients often report dissociative episodes, which may be related to an altered body awareness, and in particular to an altered awareness of the sense of agency. The sense of agency draws in part upon perceptions of being in control of our bodies and our physical movements, of being able to act upon environments. In this study, we aim to investigate whether dissociative experiences of BPD patients may be linked to an altered sense of agency and whether self-injurious actions may, through strong sensorial stimulation, constitute a coping strategy for the reduction of the distress associated with these dissociative experiences. A group of 20 BPD patients, of whom 9 presented self-harming behaviors, took part in the study and were compared with an age-matched control group of 20 healthy individuals. Sense of agency was evaluated through the Sensory Attenuation paradigm. In this paradigm, in a comparison with externally generated sensations, the degree to which perceived intensity of self-generated sensations is reduced is considered an implicit measure of sense of agency. As we expected, we found a significant difference in the perceptions of the two groups. The attenuation effect appeared to be absent in the BPD group while it was present in the control group. However, further analysis revealed that those BPD patients who engaged in self-harming behaviors presented a degree of attenuation which was similar to that of the control group. These results confirm the hypothesis that self-injurious actions constitute a coping strategy for increasing the sense of agency. We finally discuss the correlation of these experimental results with some clinical self-evaluation measures assessing dissociation, anxiety, depression, and affective dysregulation.

## Introduction

Non-suicidal self-injury (NSSI) is defined as “the deliberate, direct destruction or alteration of body tissue in the absence of conscious suicidal intent” (1). This behavior often occurs in the context of psychiatry condition, and is considered a key feature of Borderline Personality Disorder (BPD) (2, 3). BPD is characterized by disturbance in a wide range of cognitive and behavioral domains, resulting in symptoms such as intense dysphoric affect, chronic instability of mood, problematic interpersonal relationships, disturbed cognition, and recurrent self-harm. Individuals with BPD report more frequent severe and versatile NSSI compared to self-injurers without BPD. These patients report also higher rates of suicidal ideation (4).

Although research on both NSSI and BPD has increased in recent years and the prevalence and the risk factors of self-harming behaviors are now established, the function of self-injury is less well understood. Self-harming behaviors may be enacted for many purposes: seven major functions of self-injury have been aggregated in a meta-analysis study by Klonsky (5). The main functions are affect-regulation, anti-dissociation, self-punishment, interpersonal influence, anti-suicide, interpersonal boundaries, and sensation-seeking (6). Emotion dysregulation, which entails the inability to effectively regulate one’s inner emotional experiences, is thought to be a core deficit in BPD and has been considered highly associated with NSSI. Over 95% of women with BPD report engaging in NSSI for emotional relief (7–9).

Beyond this association between impaired ability to modulate affect and vulnerability for engagement in NSSI, other factors such as dissociative symptoms also appear to play an important role in NSSI. Dissociative symptoms of de-realization, depersonalization, or psychogenic amnesia are commonly found to precede the urge to engage in NSSI (10). Dissociative experiences such as distorted perceptions of feeling or action, as though one were on “automatic pilot”, have been associated with a variety of deliberate self-harm behaviors. Self-injury is viewed as a way to generate emotional and physical sensations that allows individuals to feel real and to regain a sense of self (5, 11).

Dissociation and self-harm are also linked to a number of physiological phenomena. First, a relationship between dissociation and reduced pain perception has been demonstrated. Several studies have reported that patients with BPD show reduced sensitivity to pain. Patients displayed heightened pain thresholds to stimuli involving mechanical, chemical, electrical, and thermal stimulation (12–15). Reduced sensitivity to pain has been also associated with self-harming behaviors (16, 17). One half to two thirds of these patients report hypalgesic or analgesic phenomena in association with self-injury (18). Russ and colleagues (19) reported that the absence of pain during episodes of self-injurious behavior in women with BPD was related to higher levels of anxiety, depression, dissociation, impulsiveness, trauma symptoms, and suicide attempts.

A second physiological effect of dissociation and self-harm can be related to two important components of self-awareness: the sense of body ownership and the sense of agency. Sense of body ownership refers to the feeling that different body parts belong to a unitary body (20). We know that dissociative symptoms are linked to detachment from physical experiences, including the feeling that one’s body does or does not belong to ourselves. Dissociation is thus strongly related to a distorted level of body ownership. However, the relationship between body ownership, dissociation, and BPD has not yet been systematically investigated. To our knowledge, only two studies have focused on body ownership and BPD (21, 22). Results showed a significant difference between current BPD versus remitted BPD and healthy controls in perceiving illusory ownership for an artificial limb, induced by the Rubber Hand Illusion paradigm [see, e.g., (23–25)]. Individuals with current BPD were more prone to perceive illusory ownership of the artificial limbs. This result suggests a more fragile body self-representation in BPD, compared to healthy controls and patients with BPD in remission.

Self-awareness also includes other fundamental capacities, such as the sense of agency, or the feeling of being able to control and direct one’s own actions, and through them to influence or bring about events in the external world (26). The sense of agency has been found to be impaired in some pathological conditions, such as schizophrenia (27–29). However, to our knowledge, no empirical research has evaluated the sense of agency in psychopathologies affected by dissociative symptoms and self-harm, such as BPD.

A primary aim of the present study was to investigate whether and to what extent self-harming behaviors are related to dissociative symptoms. We explored the extent to which self-harming behaviors can be considered as a coping strategy which uses strong sensory stimulation to mitigate the distress associated with dissociative experiences. We intended to evaluate the specific functions that patients attribute to NSSI behaviors and to explore the relationship between dissociative symptoms and other symptoms characterizing the BPD pathology.

A second aim of this study was to investigate whether and to what extent NSSI behaviors can modulate the sense of agency in subjects with BPD. To this aim, we measured the sense of agency in a group of patients with BPD engaging in NSSI behaviors (BPD+NSSI) and compared them to a group of BPD patients without NSSI (BPD-NSSI), and to a healthy control group. To evaluate sense of agency we made use of a specific perceptive phenomenon known as *sensory attenuation*, which demonstrates that the intensity of self-generated stimuli is perceived as attenuated in comparison with the intensity of the same stimuli generated by someone else. This phenomenon, well exemplified by the fact that one cannot tickle oneself (30, 31), demonstrates that sensorimotor predictions affect the perception of sensory stimuli. When the motor program of a voluntary action is sent to the muscles, an efferent copy of the commands is used by an internal model to predict the sensory consequences of the action. Correct predictions, based on the match between expectations and actual feedbacks, can be used to attenuate the sensory consequences of self-generated actions, which are subjectively experienced as less intense than other-generated stimuli. In other words, when predictions and outcomes match each other, afferences are not fully processed, because they do not add new information. Such phenomenon has been described in several sensory modalities [e.g., audition (32–34), vision (35, 36), tactile (24, 37–39)]. Since sensory attenuation occurs when subjects perceive a cause-effect relationship between their own actions and sensory events, this phenomenon has been proposed as an implicit marker of sense of agency (29, 40).

If the sense of agency of BPD+NSSI patients is impaired, we would expect them to show an altered sensory attenuation response when compared to BPD-NSSI and healthy controls. Additionally, we would expect that sensory attenuation results would be influenced by clinical variables such as depression, anxiety, impulsivity, and symptoms severity. Alternatively, instead of being the expression of a pathological sense of agency, an altered sensory attenuation in the BPD+NSSI group could also be explained by a low level factor, such as an increased level of tactile threshold, which has also been previously described in this clinical population (12–15).

## Material and Methods

### Participants

Twenty-two participants diagnosed with BPD according to the criteria of the DSM-5, and evaluated by the Structured Clinical Interview for DSM-IV Axis II Personality Disorders (SCID-II) (41), were enrolled in the study and signed the informed consent, together with 20 healthy adults without history of current or previous psychiatric illness. We excluded two patients with BPD from the final sample. One reported feeling unwell during the test because of a new pharmacological therapy. The other patient dropped out of the test.

The group of patients (BPD; 18 females and 2 males, range 19–49 years, *mean ± SD* = 29 ± 9.48) was matched with the control group (CTRL; 18 females and 2 males, range 21–42 years, *mean ± SD* = 25 ± 4.53) for sex and age (t_(38)_ = 1.490; *p* =.144) but not for educational level (BPD *mean ± SD* = 10.65 ± 2.98; CTRL *mean ± SD* = 16.75 ± 1.52; t_(38)_ = -8.161; *p* =.000). For both groups, exclusion criteria were: (1) substance/alcohol abuse or substance/alcohol dependence within 3 months prior to entry into the study; (2) pregnancy or breastfeeding. Furthermore, for subjects with BPD, exclusion criteria were: (1) diagnosis of schizophrenia, schizo-affective disorder, bipolar disorder, and organic mental syndrome.

Patients were divided into two groups based on the presence or the absence of NSSI behaviors: a self-harming group of patients (BPD+NSSI; N = 9) and a non-self-harming group (BPD–NSSI; N = 11). The BPD–NSSI group included three patients who had previously self-harmed but no longer engaged in NSSI behaviors.

To assess the impact of pharmacotherapy, we computed the number of medication (antidepressants (SSRI, SNRI), mood stabilizers, typical and atypical antipsychotics, benzodiazepines) and compared the two groups (BPD+NSSI vs BPD-NSSI). The pharmacological treatment did not differ between the two BPD groups (Mann-Whitney U = 44.0; *p* =.710 two-tailed) see details in **Table 1**. Recruitment and assessment of the clinical sample took place at IRCCS Centro San Giovanni di Dio Fatebenefratelli in Brescia, north Italy. The ethics committee of the IRCCS San Giovanni di Dio - Fatebenefratelli approved the experimental procedure (50, 18/07/2017).

**Table 1:** Pharmacological treatment.

Pharmacological treatment* (Molecule, quantity in mg)							
	NSSI	Antipsychotic		SSRI	SNRI	Mood stabilizer	Benzodiazepine
		Atypical	Typical				
**Pat 1**	yes	quetiapine, 300 mg		sertraline, 50 mg			triazolam, .25 mg
**Pat 2**	past			fluoxetine, 10 mg		gabapentin, 100 mg	
**Pat 3**	yes		haloperidol, 10 mg	sertraline, 100 mg		lamotrigine, 25 mg	alprazolam, 30 mg
**Pat 5**	past	aripiprazole, 30 mg					
**Pat 6**	no	risperidone, 3 mg quetiapine, 100 mg			duloxetine, 60 mg	lithium, 900 mg gabapentin, 700 mg	flurazepam, 30 mg
**Pat 8**	no					lamotrigine, 300 mg, pregabalin 300 mg	lorazepam, 2 mg
**Pat 9**	past					VASV, 1500 mg	diazepam, 20 mg
**Pat 10**	no	quetiapine, 100 mg		sertraline, 100 mg		lithium, 450 mg, VASV, 1500 mg	
**Pat 11**	yes	quetiapine, 450 mg					
**Pat 13**	yes					gabapentin, 400 mg	
**Pat 15**	yes				duloxetine, 60 mg	VASV, 500 mg	
**Pat 16**	no					VASV, 900 mg	lorazepam, 1 mg
**Pat 18**	no					VASV, 800 mg	
**Pat 19**	yes			paroxetine, 20 mg			delorazepam, 10 mg
**Pat 20**	yes					lamotrigine, 200 mg	
**Pat 22**	no	quetiapine, 100 mg				VASV, 500 mg	

NSSI, non-suicidal self-injury; SSRI, Selective serotonin reuptake inhibitors; SNRI, Selective serotonin and noradrenaline reuptake inhibitors; VASV, valproic acid-sodium valproate.

*Patients 4 and 12 are not present in this list since they were excluded from the final sample because of recent changes in the pharmacological treatment and voluntary drop out of the test. Patients 7, 14, 17, and 21 are not present because they do not undergo any pharmacological treatment.

### Experimental Procedure

**Figure 1:**
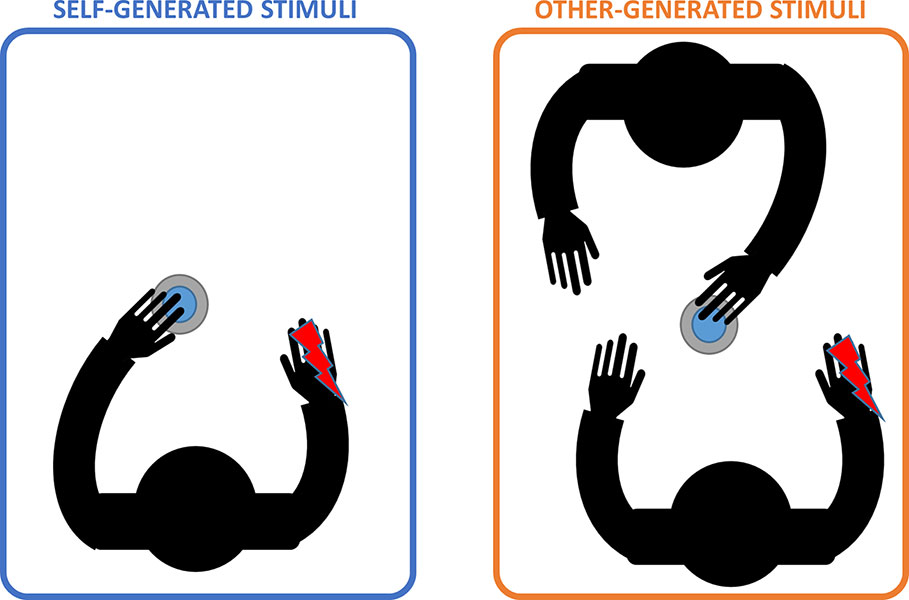
Experimental conditions. Left panel shows the “self-generated stimulation” condition (light blue) in which participants had to press the button with their left hand to deliver the stimulus (depicted as the red lighting). Right panel shows the “other-generated stimulation” condition (orange) in which participants were asked to stay still while a co-experimenter pressed the button to deliver the stimulus. Note that, in this condition, participants were asked to observe experimenter’s action.

To evaluate the *sense of agency* we used a specific research paradigm based on the Sensory Attenuation phenomenon. We asked participants to seat and place their hands on a desk. Stimuli (see details in *Electrical Stimulation* section) were randomly administered in two experimental conditions: “self-generated stimulation”, wherein subjects had to press a button with their left index finger to generate the stimulation; “other-generated stimulation”, wherein the experimenter pressed the button to generate the same stimulation (see **Figure 1**). To avoid response bias and to control for phantom sensations, catch trials (without stimulation) were randomly included and then excluded for the analysis. After each trial, subjects had to report the perceived intensity of the stimulus on a 0–7 points Likert’s Scale where 0 corresponded to “no intensity” and 7 corresponded to “very high intensity”. The experiment consisted of 20 trials of “self-generated stimulation”, 20 trials of “other-generated stimulation”, and 4 catch trials (2 self-generated and 2 other-generated catch trials) for a total of 44 stimuli.

### Electrical Stimulation

**Figure 2:**
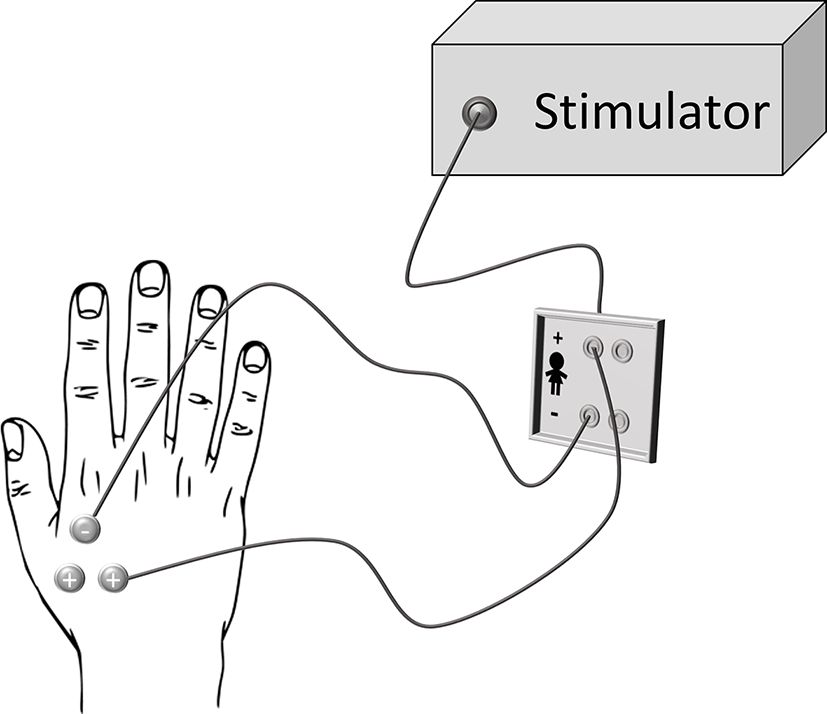
Stimulation set-up. Three electrodes were attached on the subjects’ right hand: two of them with a positive polarity and the other one with negative polarity that was also the farthest electrode from the participant’s body. For each trial, only two electrodes were engaged: the one with negative polarity and one between the other two (chosen randomly).

Transcutaneous electrical stimuli consisted in constant current square-wave pulses (Digitimer, Model DS7A) delivered to the right hand dorsum using surface bipolar electrodes attached on the flexor and abductor pollicis brevis (muscles between the metacarpal bones of the index finger and thumb). The stimulus duration was 200 μs and the stimulation intensity was adjusted according to the individual sensory threshold level (i.e., the stimulation intensity wherein participants were able to detect stimuli in the 50% of trials). The mean stimulus intensities were 2.10 ± .40 mA, range 1.46–2.64 mA for the BPD+NSSI group, 1.88 ± .77 mA, range .24–2.80 for BPD-NSSI, and 1.64 ± .39 mA, range .91–2.64 mA for the CTRL group. During the experiment the stimulation intensity was set slightly above the threshold (Stimulation intensity = intensity threshold*2.5 mA), so that participants always perceived the tactile stimulation. In order to avoid habituation, three electrodes were connected to the electrical stimulator: that one with the negative polarity was kept in the same position, while the other two with positive polarity were activated one at a time, so that participants may perceive the stimulation from two distinct part of the hand dorsum (see **Figure 2**). Each set of electrodes was activated randomly.

### Self-Report Questionnaires

At the end of the experimental procedure, participants were asked to answer to some self-report questionnaires. Each patient received a booklet including the clinical scales and received the indication to fill them out following the order of the booklet. The questionnaires were delivered after the behavioral task and returned within 3 days. The following questionnaires were included:

- *Dissociative Experiences Scale* (DES) (42) is used for the evaluation of type and severity of any dissociative aspects. It is composed of 28 items that describe the most common dissociative experiences. Subjects have to rate how frequently each of these experiences has occurred over the course of his/her life by using a 11-point Likert’s scale, which proposes a percentage from 0% at 100%.
- *Inventory of statements about self-injury* (ISAS) (43) is used for the evaluation of self-injurious behavior. The questionnaire is divided in three main sections. In the first one, the subject is questioned about the frequency and nature of self-injurious behavior throughout his/her life, proposing 12 of the most frequent self-harming behaviors (cutting, biting, burning, incising, pinching, pulling hair, severely scratching, hitting or bumping violently, interfering with wound healing, rubbing the skin against a rough surface, sticking needles, and ingesting dangerous substances). Participants are encouraged to estimate the number of times they performed each behavior. Five additional questions evaluate descriptive and contextual factors, including the age of onset, the possible experience of pain during self-injurious behavior, if it is performed when the subject is alone or with other people around, the time elapsing between impulse to injury and the effective action, and if the individual wants, or has ever wanted, to stop self-injury. The second section examine the personal motivations underlying these behaviors. It focuses on the two main factors of self-injury: interpersonal factors, which include items with regard to 8 functions (autonomy, interpersonal boundaries, influence interpersonal, bond with peers, revenge, self-care, search for sensations and test of strength, and tenacity), and intrapersonal factors, which include other 5 functions (affective regulation, anti-dissociation, anti-suicide, distress marker, and self-punishment). There are 39 items characterized by a 3-point Likert scale, where 0 = not relevant to my experience and 3 = very relevant to my experience. In the third, and last, section of the questionnaire, subjects can describe in more detail his/her own experiences regarding the functions investigated in the previous section.
- *Symptom Checklist-90-R* (SCL-90-R) (44) evaluates the presence and severity of symptoms of mental distress in the last week. The questionnaire is composed of 90 items and investigates different symptom dimensions such as somatization, obsession/compulsion, interpersonal sensitivity, depression, anxiety, hostility, phobic anxiety, paranoid ideation, psychoticism, and sleep disorders. Each item is scored on a 5-point Likert’s scale ranging from “Not at all” to “Very much”.
- *Difficulties in Emotion Regulation Scale* (DERS) (45) analyses the difficulties in regulating emotions, especially concerning negative emotions. It focuses in particular on the following dimensions: awareness and understanding of emotions, acceptance of emotions, the ability to behave in accordance with one’s goals and to regulate impulsive behavior even in the face of negative emotions, and finally the ability to use flexible strategies of emotional regulation appropriate to the context and situational demands. This scale is composed of 36 items with a 5-point Likert’s scale where 1 corresponds to “almost never” (0%–10%), 2 to “sometimes” (11%–35%), 3 to “about half the time” (36%–65%), 4 to “many times” (66%–90%) and 5 to “almost always” (91%–100%).
- *Barratt Impulsiveness Scale*, version 11 (BIS 11) (46) is used for the evaluation of impulsive traits and emotional dysregulation in the subject’s personality. The structure of the instrument allows the identification of six first-order factors and three second-order factors: first-order factors attention and cognitive instability identify *attentional impulsiveness*; perseverance and motor behavior denote *cognitive impulsiveness* and self-control and cognitive complexity specify *unplanned impulsiveness*. This tool is composed of 30 items evaluated on a 4-point Likert’s scale, where scores correspond to: 1 = never/rarely and 4 = almost always/always.
- *Beck Depression Inventory II* (BDI-II) (47, 48). It measures incidence and severity of depressive symptoms. The BDI version 2 is composed of 21 items to which the subject responds on a 4-point Likert scale (with a range from 0 to 3). Questions are based on how he/she felt in the previous two weeks about specific areas of daily life: sadness, pessimism, sense of failure, loss of pleasure, guilt, feelings of punishment, self-esteem, self-criticalness, suicidal thoughts, crying, agitation, loss of interest, indecision, sense of worthlessness, loss of energy, changes in sleeping, irritability, changes in appetite, concentration, fatigue, and loss of libido.
- *State-Trait Anxiety Inventory* (STAI-Y) (49). The questionnaire is used for the assessment of anxiety and consists of two sub-scales: T evaluates the levels of *trait anxiety*, through questions that investigate the subject about his usual mood, i.e., stable and persistent emotional state of the individual. Both scales contain 20 items, and the score is assigned on a 4-point Likert’s scale in which 1 corresponds to “not at all” and 4 to “very much”. On the contrary, S investigates *state anxiety*, i.e., questions investigate how the individual feels in the specific moment of the administration of the questionnaire, and describes his/her current moods.

### Data Analysis

#### Behavioral Analysis

Data analysis was performed using Statistica 7 software. In order to verify whether each of the three groups (BPD+NSSI, BPD-NSSI, and CTRL) could perceive the stimulation intensity as different between conditions, we first performed within-subjects analysis by comparing subjective ratings obtained in the self-generated to those of the other-generated condition through paired t-tests (2-tails). Then, in order to compare the between conditions differences in the perceived stimulation intensity between groups, we calculated an *attenuation index* (Δ) by subtracting the mean ratings of the other-generated from the mean ratings provided in the self-generated condition (Δ_n_ = S_n_ – O_n_; S: mean of the self-generated ratings of subject *n*; O: mean of the other-generated ratings of subject *n*; Δ = attenuation index of subject *n*). Therefore, an index with negative values indicated the presence of the attenuation effect (self-generated perceived as less intense as compared to other-generated stimulations), whereas an index with positive values indicated the opposite trend (other-generated perceived as less intense as compared to self-generated stimulations). The obtained *attenuation indices* (i.e., delta values) were entered in a one-way repeated measures ANOVA with group (three levels: BPD+NSSI, BPD-NSSI, and CTRL) as between subject factor. Post-hoc comparisons were performed by the Newman–Keuls test.

In order to verify whether the stimulation intensities differed among the three groups (BPD+NSSI, BPD-NSSI, and CTRL), a one-way ANOVA on the stimulation intensities was performed, and furthermore, in order to verify whether such differences may predict the between group differences in the attenuation index a one-way ANCOVA was conducted with intensity values as covariate.

Furthermore, since the groups were not match for educational level, in order to exclude that differences among Groups might be simply ascribed to the different educational levels, a one-way ANCOVA was also conducted on *attenuation indices* with group (BPD+NSSI, BPD-NSSI, and CTRL) as between subject factor and educational level as covariate.

Finally, although the two BPD groups did not differ in pharmacotherapy, to exclude that the between groups effect might be ascribed to differences in medication, we have performed a one-way ANCOVA on *attenuation indices* with group (BPD+NSSI and BPD-NSSI) as between subject factor and medication as covariate. However, it is worth noticing that the presence of pharmacotherapy is an open issue in psychiatric studies, about 90% of BPD patients receives medication with often polipharmacotherapy, despite the recommendation of the scientific society. Furthermore, it is not clear the effect of different drugs on brain functions (50, 51).

#### Questionnaires Analysis

To evaluate any difference among the three groups on the scores at the different questionnaires (DES, ISAS, DERS, BIS11, BDI-II, and STAI-Y), we ran a one-way repeated measures ANOVA with group (three levels: BPD+NSSI, BPD-NSSI, and CTRL) as between subject factor. Post-hoc comparisons were performed by the Newman–Keuls test. For the SCL-90-R and ISAS questionnaires, completed only by BPD groups we performed unpaired t-tests (2 tails). Note that questionnaires were not completed by all subjects, therefore the analysis for some questionnaires were performed on reduced samples.

#### Correlation Analysis

In order to investigate a relation between the clinical features of participants and the attenuation index, we performed Spearman correlations. To account for multiple comparisons, the significance level (*p* value) was corrected using a false discovery rate (FDR) procedure^1^ (52).

## Results

### Behavioral Results

**Figure 3:**
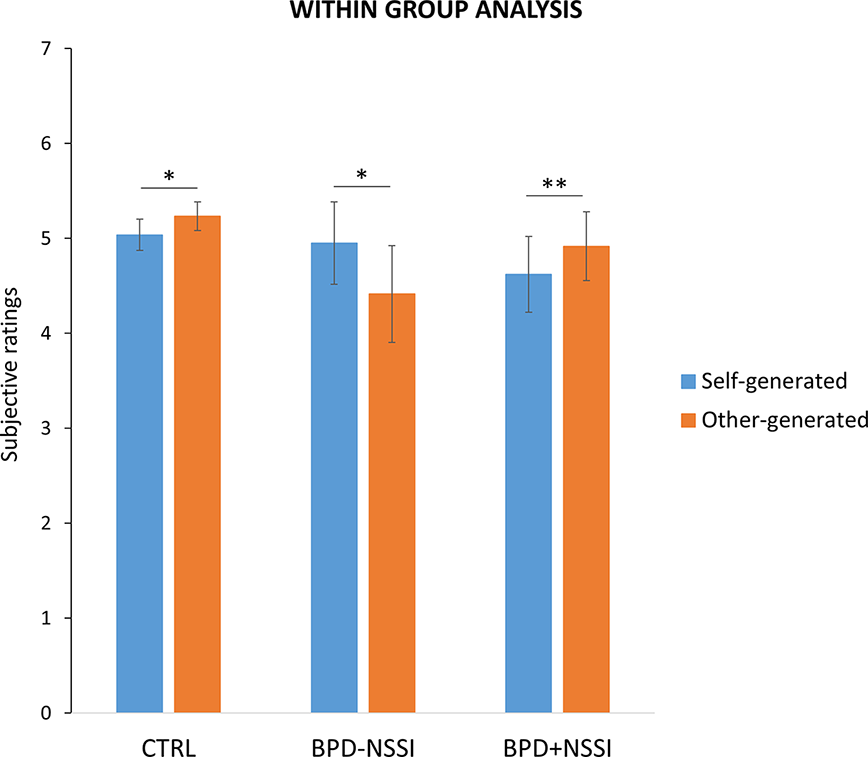
Within group analysis. Separately for each group, it is reported the significant difference between subjective ratings on the perceived painful stimuli during the two experimental conditions (i.e., self-generated stimulation in light blue and other-generated stimulation in orange). Note lower responses in self-generated compared to other-generated stimulation (i.e., sensory attenuation) in both CTRL and BPD+NSSI groups, while an opposite pattern was found in the BPD-NSSI group. Error bars indicate sem. Asterisk indicates the significant comparison (*p < .05; **p < .005).

The t-tests over the subjective ratings on the perceived stimulation intensity showed that the CTRL group, as expected, experienced the classical attenuation effect as they reported significantly less intense the self-generated (*mean ± SD* = 5.04 ± 0.75) as compared to the other-generated (*mean ± SD* = 5.23 ± 0.67) stimuli (t_(19)_ = -2.554; *p* =.019). As the CTRL group, the BPD+NSSI showed an attenuation effect, with lower ratings for the self-generated (*mean ± SD* = 4.62 ± 1.2) as compared to the other-generated (*mean ± SD* = 4.92 ± 1.08) stimuli (t_(8)_ = -3.583; *p* =.007). Interestingly, the BPD-NSSI group showed an opposite pattern compared to both controls and BPD+NSSI, reporting as significantly more intense the self-generated (*mean ± SD* = 4.95 ± 1.2) as compared to the other-generated (*mean ± SD* = 4.41 ± 1.69) stimuli (t_(10)_ = 2.460; *p* =.034) see **Figure 3**.

**Figure 4:**
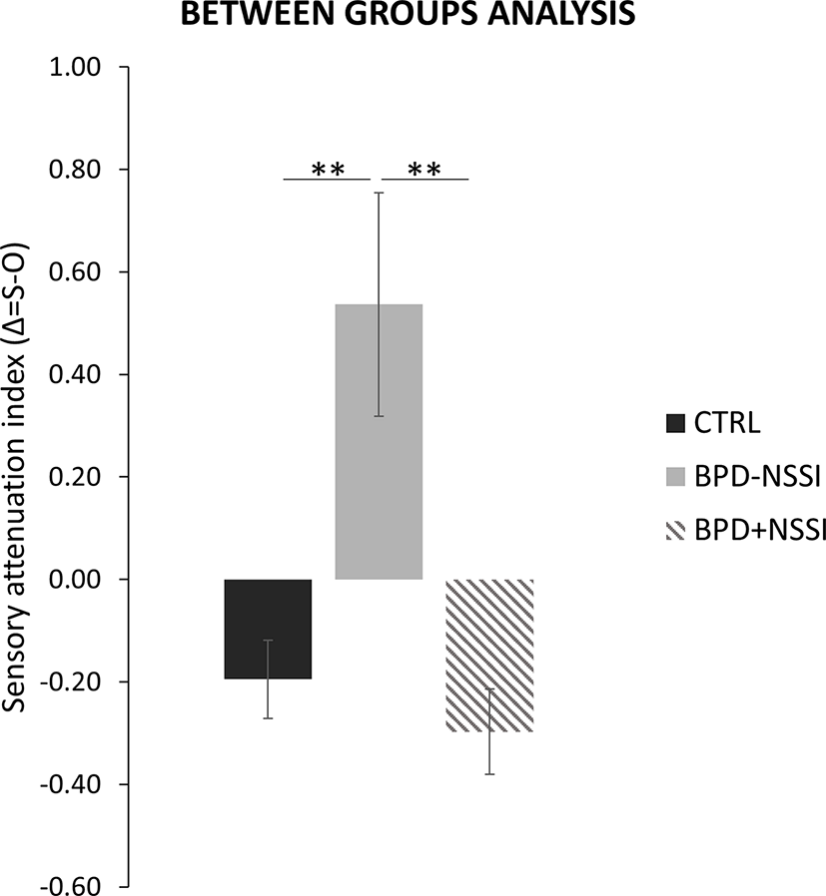
Between groups analysis. Significant differences in the sensory attenuation index between groups (CTRL in black; BPD-NSSI in gray, and BPD+NSSI in grey diagonal lines pattern). Error bars indicate sem. Asterisk indicates the significant comparison (** p < .005).

The ANOVA on the attenuation indices showed a main effect of group (F_(2,37)_ = 10.970; *p* =.0001, η^2^ =.37; power =.98) suggesting significant differences in the attenuation effect among the three groups (BPD+NSSI, BPD-NSSI, and CTRL) (see **Figure 4**). At post-hoc comparisons no difference in the attenuation effect was found between CTRL (*mean ± SD* = -.20 ± .34) and BPD+NSSI group (*mean ± SD* = -.30 ± .25) (*p* =.59). On the contrary, the BPD-NSSI group was significantly different compared to both CTRL (*p* =.0005) and BPD+NSSI group (p =.0003), showing an opposite pattern (*mean ± SD* =.54 ± .72), that is the self-generated stimuli were perceived as more intense as compared to the other-generated one.

**Figure 5:**
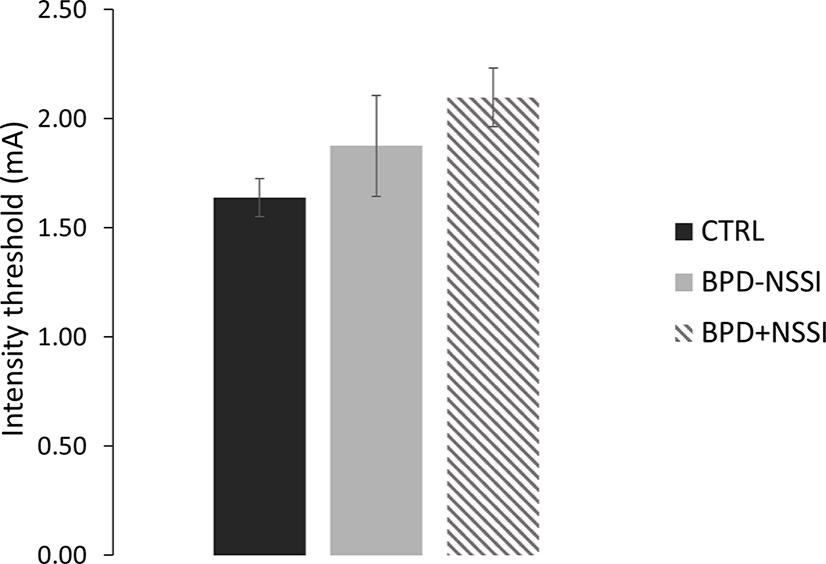
Threshold level. Separately for each group, the intensity stimulation (mA) is reported. Error bars indicate sem. (CTRL in black; BPD-NSSI in gray, and BPD+NSSI in gray diagonal lines pattern).

The ANOVA on the stimulation intensity did not show a significant effect of group (F_(2,37)_ = 2.52; *p* =.09), even if the tactile threshold was slightly different between groups. The CTRL group had the lowest threshold (*mean ± SD* = 1.64 ± .39) followed by the BPD-NSSI group (*mean ± SD* = 1.88 ± .77) and by the BPD+NSSI group that had the highest threshold (*mean ± SD* = 2.10 ± .40), see **Figure 5**.

No significant effect emerged for covariate variables medication and stimulation intensity (all *ps* >.4) whereas the covariate educational level showed a significant effect (*p* =.03). Despite this, the variable Group was always still significant after controlling for all covariate variables: medication (F_(1,17)_ = 10.099; *p* =.006), stimulation intensity (F_(2,36)_ = 10.747; *p* =.000), and educational level (F_(2,36)_ = 14.768; *p* =.000).

### Questionnaires Results

Analyses of self-report questionnaires showed that the BPD+NSSI group had the highest severity for symptomatology of the pathology (see **Table 2**).

**Table 2:** Participants’ score on self-report questionnaires.

Group scores (mean ± SD)				
Self-report questionnaires	CTRL	BPD-NSSI	BPD+NSSI	p
DES	9.98 ± 6.27	12.96 ± 10.57	31.12 ± 15.76	.0001
ISAS - Intrapersonal		10.71 ± 10.84	17 ± 7.92	.239^a^
ISAS - Interpersonal		9.57 ± 14.75	8.43 ± 3.51	.845^a^
SCL-90-R		1.59 ± 0.90	1.78 ± 0.79	.665^a^
DERS	80.65 ± 28.59	101.90 ± 31.73	131.38 ± 21.25	.0005
BIS-11	58.75 ± 10.54	64 ± 9.41	70.63 ± 9.38	.02
BDI-II	7.65 ± 8.87	16.78 ± 10.28	29.63 ± 16.74	.0002
STAI-T	35.75 ± 11.74	47.57 ± 11.72	63.40 ± 13.65	.0002
STAI-S	40.70 ± 12.79	45 ± 8.35	52.80 ± 10.89	.12

DES, Dissociative Experiences Scale; ISAS, Inventory of statements about self-injury; SCL-90, Symptom Checklist-90-R; DERS, Difficulties in Emotion Regulation Scale; BIS-11, Barratt Impulsiveness Scale, version 11; BDI-II, Beck Depression Inventory II; STAI-Y, State-Trait Anxiety Inventory; Scores of the CTRL group for SCL-90-R are not present. SCL-90-R in fact evaluates the symptoms’ severity of the pathology.

a A t-test was performed.

One-way ANOVA performed on the DES scores showed a significant main effect of group (F_(2,34)_ = 12.36; *p* =.0001) suggesting that the BPD+NSSI group gave a significantly greater score compared to both BPD-NSSI (*p* =.0003) and CTRL group (*p* =.0001). While, the DES scores were not different between BPD-NSSI and CTRL (*p* =.5).

Scores of functions of self-harming behaviors were assessed by ISAS. These scores concern the two experimental groups and results showed no significant effect of group neither in interpersonal (t_(12)_ =.199; *p* =.845) nor intrapersonal scale (t_(12)_ = 1.239; *p* =.239).

Furthermore, the ISAS allowed also a quantification of the number of self-harming behaviors. The BPD+NSSI group showed a higher number of NSSI (N= 6355) and the most frequent behavior was represented by “cutting” (N = 1027). On the contrary, the BPD-NSSI group showed a smaller number of NSSI (N = 2565) and the most frequent behavior was “interfering with wound healing” (N = 1010).

Concerning the SCL-90-R, the scale was administered only to the two BPD sub-groups since it evaluates psychopathological symptoms. We used the Global Severity Index (GSI) and detected no significant difference between the two groups (t_(14)_ =.442; *p* =.665).

The ANOVA on the DERS questionnaire, that evaluates the difficulty in emotion regulation, showed a significant main effect of group (F_(2,35)_ = 9.5; *p* =.0005), suggesting an higher score in the BPD+NSSI group compared to both BPD-NSSI (*p* =.02) and CTRL group (*p* =.0006). On the contrary the difference between BPD-NSSI and CTRL group was marginally significant (*p* =.08).

Regarding BIS-11 questionnaire, evaluating the impulsiveness level, we observed a significant effect of group (F_(2,33)_ = 4.08; *p* =.02) indicating that the BPD+NSSI group had a significantly higher score compared to the CTRL group (*p* =.03) and not compared to BPD-NSSI group (*p* =.15). However, even if the BIS-11 score of the BPD-NSSI group was higher than CTRL group, this did not reach the significance level (*p* =.25).

Results on the BDI-II, evaluating the depression symptoms, showed a main effect of group (F_(2,34)_ = 11.1; *p* =.0002), suggesting an higher score for the BPD+NSSI group compared to both BPD-NSSI (*p* =.01) and CTRL group (*p* =.0003). Between BPD-NSSI and CTRL there was a trend (*p* =.07) suggesting greater depression symptoms in the pathological group.

Regarding anxiety, ANOVA on STAI-T scores showed a main effect of group (F_(2,29)_ = 11.3; *p* =.0002) indicating an higher score for the BPD+NSSI group compared to both BPD-NSSI (*p* =.01) and CTRL group (*p* =.0004). While, between BPD-NSSI and CTRL only a trend was observed (*p* =.06).

Conversely, no significant effect emerged from the ANOVA over the STAI-S (F_(2,30)_ = 2.23; *p* =.12).

### Correlation Results

No significant correlations were observed between questionnaires and sensory attenuation index (always *p* >.05).

## Discussion

The present study was aimed at investigating the role of dissociation and the sense of agency in individuals with BPD with and without NSSI behaviors. To this aim we exploited the well-known Sensory Attenuation phenomenon, considered to be an implicit measure of sense of agency (26).

Our initial hypothesis was that the BPD+NSSI group would show higher dissociative symptoms. The results confirmed the hypothesis that dissociation is related to NSSI behavior. Coherently with our hypothesis, BPD with NSSI showed higher dissociative symptoms in comparison with both BPD without NSSI and healthy controls. The relationship between dissociation and NSSI seems also to be confirmed by the number of NSSI which is extremely higher in the BPD+NSSI group.

The clinical functions of self-harm are manifold: affect-regulation, anti-dissociation, self-punishment, interpersonal influence, anti-suicide, interpersonal boundaries, and sensation-seeking (6). Furthermore, more recent research has identified in attentional focusing a possible mediator between BPD and self-harm (53). One of the possible hypothesis is that by inducing physical pain, patients with dissociative symptoms may regulate feelings of distress related to dissociation, such as a sense of loss of control, an estrangement from reality, and experiences of numbness (5, 54, 55). However, since the highest percentage of dissociation was found in our sample of BPD+NSSI, this suggests that the temporal relief afforded by NSSI behaviors is not effective for the long-term reduction or mitigation of dissociative symptoms. Although dissociation and NSSI are linked, their temporal relationship remains unclear. Patients’ clinical reports suggest that states of dissociation precede acts of NSSI. However, it is also possible that some states of dissociation may be the result of NSSI behaviors (56). Further investigation will be needed in order to understand the causal relationship between NSSI behaviors and dissociative symptoms.

The second aim of the study was to evaluate the sense of agency in BPD with and without NSSI behaviors. We expected that BPD+NSSI would show less sensory attenuation than BPD-NSSI and controls. In other words, we expected that they would be unable to discriminate between self- and other-generated stimuli and would therefore show less sensory attenuation in self-generated stimulation than controls.

The data revealed an unexpected result for both clinical groups (BPD+NSSI and BPD-NSSI). Indeed, the BPD+NSSI group did not differ from the CTRL group and they showed a usual pattern of sensory attenuation. In contrast, the BPD-NSSI group showed sensory attenuation with a reverse pattern, perceiving self-stimulation as more intense than other-generated stimulation.

These findings might suggest a counterintuitive effect of NSSI behaviors. We speculate that the NSSI behaviors may generate a sense of agency by virtue of having used an active strategy to overcome an aversive internal state. From the self-reports of NSSI patients, we know that one result produced by cutting is a modification of the sense of unreality, of being unreal or indeed of being nothing at all, which precedes the act. The act of cutting appears to enable a new set of emotional and physical sensations which allow the individual to feel alive again. We may therefore hypothesize that these subjective sensations may also be linked to a renewed sense of agency, of being an individual who is capable of taking action in and on his/her environment, and who can plan and carry out intentional actions. From this standpoint, it is plausible that cutting may also contribute to re-establishing awareness of physical agency. This could account for the evaluation of the BPD+NSSI group as similar to healthy controls in the paradigm of sensory attenuation.

It is important to note that this difference between BPD+NSSI and BPD-NSSI cannot be attributed to differences in perceptive thresholds. Even though both BPD groups showed a significantly higher threshold than the CTRL group, such difference was not statistically significant and did not predict the differences in sensory attenuation among groups. This result is in line with previous studies which suggest a specific sensory perception in pain domain in patients with BPD, but no alteration in tactile proprioceptive perception compared to healthy controls (21, 57).

Furthermore, the group differences in the sensory attenuation remained significant even when controlling for educational level which appeared to be significantly different among groups. Whether it could be argued that education could be a possible confounder, it could be noted that a lower level of education compared with general population is a common feature in psychiatric patients (58) and it is strictly linked with psychopathology. Future studies with larger sample will allow to better clarify the impact of education.

Further clarification is also required for the anomalous results of the BPD group who do not engage in NSSI behaviors. Currently, we can propose no hypotheses or links to other clinical aspects of BPD. DES scores do not explain differences on the sensory attenuation effect. However, a larger sample could perhaps generate further insights into the relationship between dissociative aspects and sense of agency.

Consistently with previous findings, our data suggest that BPD patients who enact NSSI behaviors present a more complex psychiatric profile than those patients who do not engage in NSSI behaviors (4, 21). Along with dissociative symptoms, BPD+NSSI exhibited higher score of anxiety disorders, depression, emotion dis-regulation and impulsivity, and dysfunctional coping strategies. However, clinical features do not show any relation with the sensory attenuation phenomenon and thus with the sense of agency.

This study has a number of limitations which must be addressed. One important limitation is the low number of patients involved. The small size of the two clinical groups (9 BPD+NSSI and 11 BPD-NSSI) could have affected the statistical power of our analysis and did not allow us to find significant correlations with most of the symptoms’ measurements. The absence of cognitive measures represents another main limit. In recent years, increasing literature focused on the role of neurocognitive deficits in the development of BPD with growing evidence pointing to cognitive deficits in Executive Functions (53, 59, 60). Although some evidence suggests that NSSI patients may exhibit more severe Executive Functions deficits, several studies support the idea that cognitive neuropsychological deficits may represent one of the core aspects of BPD. Certainly, this aspect has to be considered in upcoming research.

Future studies would benefit from distinguishing clearly between trait aspects and state aspects of dissociation. This would facilitate a more fine-grained understanding of the role played by dissociative states and by sense of agency in NSSI behaviors. The current study evaluated only trait aspects of dissociation. We are therefore unable to establish clear temporal and causal relationships between NSSI and dissociation. Evaluation of state traits of dissociation could possibly enhance understanding of both antecedents and consequences of acts of NSSI and their relationship with the sense of agency.

## Data Availability Statement

The datasets generated for this study are available on request to the corresponding author.

## Ethics Statement

The studies involving human participants were reviewed and approved by IRCCS San Giovanni di Dio - Fatebenefratelli (50, 18/07/2017). The patients/participants provided their written informed consent to participate in this study.

## Author Contributions

LC, CF, and FG designed the research. DH and RR performed research. DH, RR, and CF analyzed data. DH and CF prepared figures. RR recruited patients and performed psychological assessment. All authors wrote the manuscript.

## Funding

This work has been funded by RILO 2018 (Linea A) - CDD 17/07/2018 to LC, by MIUR-SIR 2014 grant (RBSI146V1D) to FG, and by the San Paolo Foundation 2016 grant (CSTO165140) to FG.

## Conflict of Interest

The authors declare that the research was conducted in the absence of any commercial or financial relationships that could be construed as a potential conflict of interest.

## Footnotes

1 The Benjamini-Hochberg FDR procedure allows to control the fact that some p-values may happen by chance. It consists in putting all p-values in ascending order, assigning them ranks and applying a specific formula (I/T)P where I is the individual p-value’s rank, T is the total number of performed tests, and P indicates the percentage of false discovery rate.
